# B cell repopulation kinetics after rituximab treatment in ANCA-associated vasculitides compared to rheumatoid arthritis, and connective tissue diseases: a longitudinal observational study on 120 patients

**DOI:** 10.1186/s13075-017-1306-0

**Published:** 2017-05-18

**Authors:** Jens Thiel, Marta Rizzi, Marie Engesser, Ann-Kathrin Dufner, Arianna Troilo, Raquel Lorenzetti, Reinhard E. Voll, Nils Venhoff

**Affiliations:** Department of Rheumatology and Clinical Immunology, Medical Center – University of Freiburg, Faculty of Medicine, Hugstetterstrasse 55, 79106 Freiburg, Germany

**Keywords:** ANCA, B lymphocyte, Rituximab, Repopulation, Hypogammaglobulinemia

## Abstract

**Background:**

B cell depletion with rituximab (RTX) is approved for treatment of rheumatoid arthritis (RA) and ANCA-associated vasculitides (AAV). Recently, RTX has been shown to be effective in AAV maintenance therapy, but an optimal RTX treatment schedule is unknown and the time to B cell repopulation after RTX has not been studied.

**Methods:**

Retrospective single-center analysis of B cell repopulation in patients with AAV, RA or connective tissue disease (CTD) treated with RTX.

**Results:**

Beginning B cell repopulation within the first year after RTX treatment was observed in 93% of RA and 88% of CTD patients. Only 10% of patients with granulomatosis with polyangiitis (GPA) and microscopic polyangiitis (MPA) and no patient with eosinophilic granulomatosis with polyangiitis (EGPA) showed B cell repopulation within this time. Median time of B cell depletion was 26 months in GPA/MPA, and 21 months in EGPA compared to 9 months in RA, and 8 months in CTD (*p* < 0.0001). In 25 AAV-patients B cell depletion lasted for at least 44 months. There was a significant decline in serum immunoglobulin concentrations in GPA/MPA patients, but not in patients with RA or CTD. Significantly more GPA/MPA patients developed hygogammaglobulinemia (IgG <7 g/L) compared to patients with RA or CTD.

**Conclusions:**

In contrast to RA and CTD, in AAV RTX induces long-lasting depletion of B cells that is associated with decreased antibody production. This observation points toward potential defects in the B cell compartment in AAV that are unmasked by immunosuppressive treatment and has important implications for the design of maintenance treatment schedules using RTX.

## Background

B lymphocytes are central to the pathogenesis of autoimmune diseases by production of autoantibodies and pro-inflammatory cytokines. B cells are also crucial to the maintenance of a proper immune defense by antigen presentation and generation of protective antibodies. B cell targeting with rituximab (RTX) depletes all CD20-carrying B cell subpopulations but does not affect bone marrow B cell precursors and antibody-producing plasma cells as these cells are devoid of surface CD20 expression. Replenishment of the peripheral B cell compartment arising from RTX-resistant early B cell precursors and persistent production of protecting antibodies by long-lived plasma cells make RTX a relatively safe treatment. Indeed, it has been reported that B cell repopulation starts with transitional B cells 6 to 9 months after RTX treatment in patients with rheumatoid arthritis (RA) [1] and most studies do not describe a clinically relevant decrease in immunoglobulin concentrations [2–5]. In connective tissue diseases (CTD) B cell proliferation and immunoglobulin production are often increased and a rapid repopulation after RTX treatment has been reported [6]. Rituximab is approved for the treatment of RA and recently for induction therapy of ANCA-associated vasculitides (AAV). As AAV are frequently relapsing diseases and maintenance treatment is challenging, repeated RTX infusions are increasingly used as maintenance treatment [7]. Importantly, in AAV the widely used 6-monthly RTX-infusion intervals are based on data obtained in RA patients, but without clinical studies supporting this schedule in AAV. We observed a clinically relevant decline in serum immunoglobulin concentrations in AAV patients treated with RTX [8] indicating a less robust B cell compartment in AAV and monitoring of immunoglobulins is now part of the EULAR guidelines for RTX-treated AAV [9]. Furthermore, we recently described that in some AAV patients B cell repopulation can be delayed or completely absent even years after RTX treatment [10]. Here, we compare kinetics of B cell repopulation after RTX treatment in AAV, RA, and connective tissue diseases. Our results have implications on the design of RTX-retreatment schedules in AAV and other autoimmune diseases.

## Methods

The study is a single-center retrospective analysis of patients attending the Department of Rheumatology and Clinical Immunology at the University Medical Center Freiburg. Inclusion criteria to the study were: RTX treatment and a follow-up of at least 12 months. All patients of AAV, RA, and CTD patient cohorts at the University Medical Center Freiburg were screened and included into the study if they met the inclusion criteria. One hundred twenty patients were enrolled and monitored 3-monthly. Sixty-six patients had a diagnosis of AAV with 55 patients classified as granulomatosis with polyangiitis (GPA) or microscopic polyangiitis (MPA) and 11 patients classified as eosinophilic granulomatosis with polyangiitis (EGPA) according to current criteria [11, 12]. Patients were compared to 35 RA and 19 CTD patients. Peripheral B cell counts were measured using a whole blood staining by flow cytometry with a FACS Canto II (BD Biosciences, San Jose, CA, USA) and PE-Cy7-conjugated anti-CD19 (clone: J3.119; Beckman Coulter, Brea, CA, USA). Results were expressed both as absolute cell counts and relative percentages. B cell depletion was defined as peripheral CD19^+^ B cells ≤ 1/μl or ≤ 0.1% of total lymphocytes. Incomplete B cell repopulation was defined as a peripheral B cell count between 5 and 69 B cells/μl and > 0.5% B lymphocytes. Complete B cell repopulation was defined as >70 B cells/μl. A timely B cell repopulation was defined by occurrence of peripheral B cells within 12 months. For assessing the time of B cell depletion we used the time from RTX administration till: (a) the last time point of B cell measurement with ≤5 B cells/μl and B cells ≤0.5% (no further measurements or repopulation at the time point of the next B cell measurement) or (b) the time point of RTX re-treatment and no prior repopulation of B cells. Thus, patients re-treated with RTX because of recurring disease activity were censored at the time of re-treatment and if B cells were still < 5/μl the time until re-treatment was regarded as the time of B cell depletion. Statistics were done using GraphPad Prism 5 (GraphPad Software, Inc., La Jolla, CA, USA) and two-sided chi-square test, Wilcoxon analysis and Student’s *t* test. Data are reported in median and interquartile range (IQR) if not stated otherwise. The log rank test and the Spearman correlation coefficient were used to analyze for a potential correlation of cyclophosphamide sum dose and time of B cell depletion.

## Results

Patients’ characteristics are summarized in Table 1. Cumulative doses of prior cyclophosphamide (CYC) treatment were higher in patients with AAV or CTD than in RA patients, but not different between patients with AAV and CTD. The percentage of patients that were treated with a concomitant immunosuppressive therapy in addition to RTX was not different between the groups. While GPA and EGPA patients were treated with higher glucocorticoid dosage before RTX administration compared to RA patients, there was no difference in glucocorticoid dosage between GPA/EGPA and CTD patients, and RA and CTD patients. Furthermore, 6 months after rituximab treatment, mean glucocorticoid dosage was below 10 mg/d for all patient groups (RA 5.6 mg/d, GPA 6.4 mg/d, CTD 7.2 mg/d), except for EGPA (11.5 mg/d) and the cumulative glucocorticoid dosage was not different between GPA/MPA, RA, and CTD patients. Only EGPA patients had higher prednisone sum doses.

**Table 1:** Patients’ characteristics

	ANCA-associated vasculitides		Other autoimmune diseases	
	GPA and MPA	EGPA	RA	CTD
Patients, n	55	11	35	19
Female/male, n	24/31	4/7	20/15	15/4
Age at RTX treatment, years	56.2 (47.6–66.5)	54.4 (37.0–62.2)	59.0 (51.0–66.0)	53.0 (46.0–69.0)
Body mass index	26.6 (21.9–29.4)	25 (19.6–28.2)	26.6 (21.5–30.4)	24.1 (20.2–27.5)
Autoantibodies	ANCA (100%)	ANCA (36%)	RF (94%)	ANA (100%)
	anti-PR3 (89%)	anti-PR3 (0%)	anti-CCP (94%)	
	anti-MPO (9.1%)	anti-MPO (36%)		
Rituximab (one course)				
RTX dose, g	2.0 (2.0–2.86)	2.0 (2.0–2.0)	2.0 (1.0–2.0)	2.0 (2.0–3.0)
2 × 1000 mg, n (%)	41 (75)	11 (100)	27 (77)	15 (79)
4 × 375 mg/m^2^, n (%)	14 (25)	0 (0)	0 (0)	1 (3)
Other regimen, n (%)	0 (0)	0 (0)	8 (23)	3 (16)
Rituximab (two courses)				
patients, n (%)	44 (80)	8 (73)	22 (63)	8 (42)
Mean time (± SEM) between first and second RTX course (months)	25.5 (±3.1)	19.3 (±4.8)	20.5 (±3.6)	19 (±3.9)
Cyclophosphamide				
cumulative dose (g)	8.8 (3.5–25.0)^†^	7.1 (0.0–9.8)^†^	0.0 (0.0–0.0)	4.5 (0.0–7.7)^†^
Min. to max. (g)	0.0–216.0	0.0–12.0	0.0–17.5	0.0–10.0
Pat. without CYC, n (%)	8 (14.5)	3 (27.3)	30 (85,7)	6 (31.6)
Current (previous) concomitant immunosuppressive treatment, n				
No DMARD, n	13 (19)	0 (1)	7 (0)	1 (2)
PRED, n	55 (55)	11 (11)	32 (35)	18 (19)
cumulative dose (g)	2.28 (1.72–2.66)^**^	3.63 (2.88–4.62)	1.8 (1.73–2.52)^**^	2.7 (1.8–3.24)^*^
Min. to max. (g)	0.46-3.73	2.22–5.02	0–5.85	0–10.8
MTX, n	10 (22)	1 (5)	16 (29)	3 (6)
cumulative dose (g)	0.78 (0.78–0.78)	0.78 (0.78–0.78)	0.78 (0.39–0.88)	0.78 (0.78–0.78)
Min. to max. (g)	0.78–0.78	0.78–0.78	0.39–1.3	0.78–0.78
LEF, n	11 (13)	0 (1)	5 (16)	2 (3)
cumulative dose (g)	7.3 (7.3–7.3)	0	7.3 (5.5–7.3)	7.3 (7.3–7.3)
Min. to max. (g)	7.3-7.3	0	3.7-7.3	7.3-7.3
AZA, n	15 (15)	9 (8)	0 (1)	2 (9)
cumulative dose (g)	36.5 (36.5–54.8)	54.7 (45.6–63.9)	0	36.5 (36.5–36.5)
Min. to max. (g)	27.4–54.8	36.5–73	0	36.5–36.5
MMF, n	6 (3)	1 (1)	0 (2)	8 (11)
cumulative dose (g)	35.6 (35.6–71.2)	35.6 (35.6–35.6)	0	73 (66.2–73)
Min. to max. (g)	35.6–71.2	35.6–35.6	0	36.5–
HCQ, n	0 (1)	0 (0)	8 (13)	9 (9)
cumulative dose (g)	0	0	73 (73–73)	73 (73–73)
Min. to max. (g)	0	0	73–73	73–73

Data are reported in median and IQR if not stated otherwise. Cumulative doses for immunosuppressive agents other than CYC were calculated for each individual for the period of 12 months after the first RTX course

*Abbreviations*: *ANA* antinuclear antibodies, *ANCA* anti-neutrophil cytoplasmic antibodies, *AZA* azathioprine, *CCP* cyclic citrullinated peptide, *CTD* connective tissue disease, *CYC* cyclophosphamide, *EGPA* eosinophilic granulomatosis with polyangiitis, *GPA* granulomatosis with polyangiitis, *HCQ* hydroxychloroquine, *LEF* leflunomide, *Min. to max.* minimal to maximal dose, *MMF* mycophenolate mofetil, *MPA* microscopic polyangiitis, *MPO* myeloperoxidase, *MTX* methotrexate, *PR3* proteinase 3, *PRED* prednisone, *RA* rheumatoid arthritis, *RF* rheumatoid factor, *RTX* rituximab

^†^ *p* < 0.05 compared to RA; ^*^ *p* < 0.05 compared to EGPA; ^**^ *p* < 0.001 compared to EGPA

**Fig. 1:**
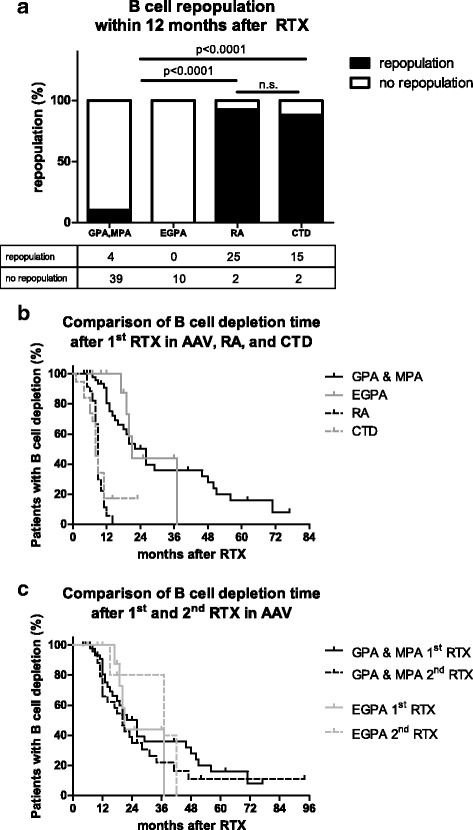
B cell repopulation and time of B cell depletion after rituximab. B cell repopulation within the time period of 12 months was detectable in approximately 90% of the patients with RA or CTD but in less than 10% of the AAV patients (**a**). Median time of B cell depletion after RTX was significantly prolonged in patients with GPA and MPA or EGPA compared to patients with RA or CTD (**b**). After a second RTX course B cell depletion time and repopulation kinetics were similar compared to the first RTX treatment course in AAV patients (**c**). *Abbreviations*: *AAV* ANCA-associated vasculitides, *CTD* connective tissue disease, *EGPA* eosinophilic granulomatosis with polyangiitis, *GPA* granulomatosis with polyangiitis, *MPA* microscopic polyangiitis, *n.s.* not significant, *RA* rheumatoid arthritis, *RTX* rituximab

All patients with RA or CTD showed a B cell repopulation within the total observation period compared to only 33 AAV patients (50%) (*p* < 0.0001). B cell repopulation within the first 12 months after RTX therapy was observed in 93% of RA patients and 88% of CTD patients compared to only 10% in GPA and 0% in EGPA patients (*p* < 0.0001) (Fig. 1a). B cell repopulation was not only delayed but also incomplete in most AAV patients compared to patients with RA or CTD. Only five AAV patients (7.5%) reached normal B cell numbers at a median of 17 months (IQR 14–20) after RTX (data not shown). Regarding the kinetics of B cell repopulation the median time of persistent depletion was 26 months in GPA/MPA and 21 months in EGPA compared to 9 months in RA and 8 months in CTD (*p* < 0.0001) (Fig. 1b). After a first single RTX treatment consisting either of four infusions in weekly intervals or two infusions of 1 g within 2 weeks, B cell depletion persisted for more than 24 months in 25 AAV patients (38%) (mean time of depletion 44 months +3.668 SEM). In the longest available follow-up, persisting B cell depletion after one RTX treatment cycle was observed for more than 5 years in six AAV patients and in one patient for even 8 years after RTX. No correlation was found between CYC sum dose and time of B cell depletion in AAV patients (r = 0.08864, *p* = 0.5199). In four (36%) of the 11 AAV patients without any CYC treatment B cell depletion time was severely prolonged with a range from 17 to 62 months. As mentioned, the percentage of patients that were treated with a concomitant immunosuppressive therapy was not different between the groups, but before and after administration of RTX azathioprine (AZA) and methotrexate (MTX) were the most commonly used immunosuppressive drugs in GPA and EGPA, while it was azathioprine (AZA) and mycophenolate (MMF) in CTD, and MTX, sulfasalazine (SSZ) and leflunomide (LEF) in RA. Neither maintenance therapy with MTX, nor with MMF, SSZ, or LEF prolonged the time of B cell depletion in the different patient groups. Furthermore, cumulative MTX, LEF, and MMF dosages after RTX were not different between the patient groups (Table 1). B cell depletion was significantly longer in AAV patients treated with AZA (21 months, IQR 13.5–64.25) as concomitant therapy compared to those treated with other immunosuppressants (15.5 months, IQR 11–26.75) (*p* = 0.0485). Interestingly, in CTD patients the use of AZA did not increase the time of B cell depletion. Furthermore, the cumulative AZA dosage after RTX was not different between AAV and CTD patients.

**Fig. 2:**
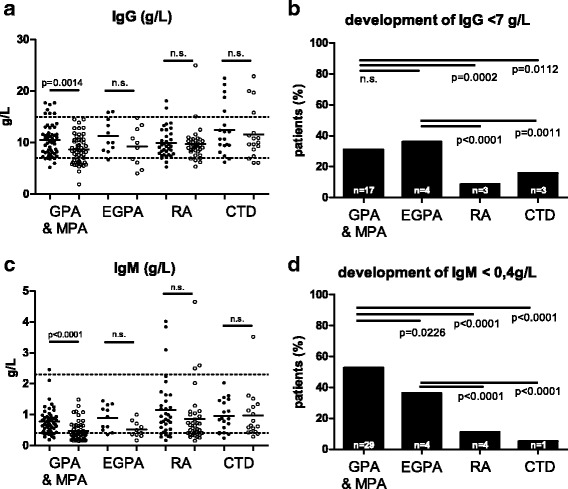
Serum immunoglobulin concentrations before and after RTX treatment and frequency of newly developed hypogammaglobulinemia. Serum IgG and IgM concentrations significantly dropped after RTX treatment compared to baseline in patients with GPA and MPA, but not in RA and CTD (**a**, **c**). Compared to patients with RA or CTD significantly more patients with GPA and MPA or EGPA developed new hypogammaglobulinemia of the isotypes IgG (**b**) and IgM (**d**). *Abbreviations*: *CTD* connective tissue disease, *EGPA* eosinophilic granulomatosis with polyangiitis, *GPA* granulomatosis with polyangiitis, *IgG* immunoglobulin G, *IgM* immunoglobulin M, *n.s.* not significant, *MPA* microscopic polyangiitis, *RA* rheumatoid arthritis

Forty-four patients with GPA/MPA and eight patients with EGPA received a second course of RTX because of relapse or as maintenance therapy. In AAV patients that required retreatment with RTX the latency between first and second RTX administration was 25 months (mean; ± 2.8 months). There was no difference in time to repopulation between the first and the second RTX cycle (Fig. 1c). Immunoglobulin production was affected by RTX treatment with a significant decline in immunoglobulin (Ig)G, IgA and IgM serum concentrations in GPA/MPA patients, but not in patients with RA or CTD (Fig. 2a and c). Hypogammaglobulinemia with IgG below the normal limit was significantly more frequent in GPA/MPA and EGPA compared to RA and CTD (Fig. 2b and d). Seven patients (10.6%) with AAV required immunoglobulin replacement therapy at least once during follow-up. GPA/MPA patients with a long-lasting B cell depletion (>24 months) were more likely to develop hypogammaglobulinemia than GPA/MPA patients with B cell depletion time of up to 12 months (30% vs. 0%, *p* < 0.0001).

## Discussion

B cell depletion is effective in many autoimmune diseases. When B cell-targeted therapy is discontinued the effect wears off [13, 14] and therefore 6-monthly re-treatment schedules are common clinical practice. B cell repopulation kinetics are of crucial importance for the design of rituximab re-treatment schedules, but have to date not been systematically compared in different autoimmune diseases. Our study demonstrates for the first time the highly divergent B cell repopulation kinetics in ANCA-associated vasculitides compared to rheumatoid arthritis and connective tissue diseases. Based on data in RA patients [1] a timely start of B cell repopulation is expected within the first 9 to 12 months after RTX treatment. We observed a starting B cell repopulation within the first year after RTX therapy in 93% of RA patients and 88% of the CTD patients compared to only 10% in GPA/MPA and 0% in EGPA. While all in all data on repopulation kinetics after administration of RTX are scarce in patients with autoimmune disease, some studies reported on patients with prolonged B cell depletion, but the frequency of these patients was low [15, 16, 13, 17]. A publication by Jones et al. [13] reported a repopulation of peripheral B cells 11 months after RTX treatment in only about 50% of the patients. Unfortunately, the lack of a consensus for the definition of depletion and especially repopulation complicates the interpretation of the existing data. A further confounder regarding B cell repopulation after RTX are different concomitant or prior immunosuppressive therapies in the patients groups. CYC can effectively deplete B cells, but we found no correlation between CYC sum dose and the time of B cell depletion. Furthermore, a severely prolonged B cell depletion was also observed in AAV patients never treated with CYC before. B cell numbers after RTX treatment were assessed in a large, randomized trial of RTX in RA patients. In this study patients were treated with RTX as monotherapy or in combination with MTX or cyclophosphamide. A small subset of patients in this study showed a prolonged B cell depletion at week 104. Of interest, patients with a prolonged B cell depletion were either treated with RTX monotherapy of RTX/MTX but not RTX/CYC [18]. We found no hints that concomitant immunosuppressive therapy with MTX, MMF, and glucocorticoids affected the time to repopulation in our patients. Interestingly, in AZA-treated patients time to repopulation was prolonged in AAV patients, but not in patients with CTD (while the cumulative AZA dosage after RTX was not different between AAV and CTD patients). Different studies have shown a profound effect of azathioprine on the lymphoid cell lineage [19, 20]. Azathioprine is known for potential myelotoxicity, and variabilities in inter-individual or inter-diseases thiopurine metabolism may influence myelotoxicity. Thiopurine methyltransferase (TPMT), inosine triphosphate pyrophosphatase (ITPA), or NUDT15 gene variants may influence the effect of AZA on B cell precursor cells in the bone marrow of AAV patients [21]. As AZA prolonged B cell depletion time only in AAV patients but not in patients with CTD, it is unlikely that AZA-related myelotoxicity is a general mechanism leading to prolonged B cell depletion time. Furthermore, even among the AAV patients, there were patients with long-term B cell depletion that never received AZA. Therefore, AZA is probably a contributing factor slowing B cell repopulation down, but very likely not the main reason for the long-term B cell depletion in AAV patients. As the patients of this study were from a single center in southern Germany and of predominantly Caucasian origin, a specific genetic background leading to alterations in stromal cells or B cell progenitors that are unmasked by B cell depletion are potential reasons for our observation of a prolonged B cell depletion in AAV. The prolonged B cell depletion time in AAV is of clinical importance as the frequency of newly developed hypogammaglobulinemia - even after only one course of RTX treatment - was significantly higher in AAV patients compared to patients with RA or CTD. Thus, especially in AAV serum Ig concentrations should be monitored closely during follow-up. Our data suggest, that the influence of rituximab on the B cell compartment differs greatly between AAV and other autoimmune diseases. This has to be taken into account when designing RTX re-treatment schedules.

## Conclusions

B cell repopulation kinetics after rituximab treatment differ significantly between patients with rheumatoid arthritis, connective tissue diseases, and ANCA-associated vasculitides. In AAV RTX induces a very long-lasting depletion of B cells, pointing toward an altered B cell maturation capacity in this disease. Our observation is of immediate clinical importance, as it affects the tailoring of RTX re-treatment schedules. Furthermore, as the prolonged B cell depletion leads to decreased antibody production, regular measurements of serum immunoglobulin concentrations after RTX treatment are indicated in AAV.

## Abbreviations

AAV: ANCA-associated vasculitides
AZA: Azathioprine
CTD: Connective tissue disease
CYC: Cyclophosphamide
EGPA: Eosinophilic granulomatosis with polyangiitis
GPA: Granulomatosis with polyangiitis
Ig: Immunoglobulin
IQR: Interquartile range
LEF: Leflunomide
MMF: Mycophenolate mofetil
MPA: Microscopic polyangiitis
MTX: Methotrexate
RA: Rheumatoid arthritis
RTX: Rituximab
SSZ: Sulfasalazine

## References

[1] Roll P, Palanichamy A, Kneitz C, Dorner T, Tony H-P (2006). Regeneration of B cell subsets after transient B cell depletion using anti-CD20 antibodies in rheumatoid arthritis. Arthritis Rheum.

[2] Popa C, Leandro MJ, Cambridge G, Edwards JCW. Repeated B lymphocyte depletion with rituximab in rheumatoid arthritis over 7 yrs Rheumatology. 2007;46(4):626–30. 10.1093/rheumatology/kel393 17189244

[3] van Vollenhoven RF, Emery P, Bingham CO, Keystone EC, Fleischmann R, Furst DE (2010). Long-term safety of patients receiving rituximab in rheumatoid arthritis clinical trials. J Rheumatol.

[4] Keogh KA, Ytterberg SR, Fervenza FC, Carlson KA, Schroeder DR, Specks U (2006). Rituximab for refractory Wegener's granulomatosis. Am J Respir Crit Care Med.

[5] Holle JU, Dubrau C, Herlyn K, Heller M, Ambrosch P, Noelle B (2012). Rituximab for refractory granulomatosis with polyangiitis (Wegener's granulomatosis): comparison of efficacy in granulomatous versus vasculitic manifestations. Ann Rheum Dis.

[6] Albert D, Dunham J, Khan S, Stansberry J, Kolasinski S, Tsai D (2008). Variability in the biological response to anti-CD20 B cell depletion in systemic lupus erythaematosus. Ann Rheum Dis.

[7] Pagnoux C, Guillevin L (2015). French Vasculitis Study G, investigators M. Rituximab or azathioprine maintenance in ANCA-associated vasculitis. N Engl J Med.

[8] Venhoff N, Effelsberg NM, Salzer U, Warnatz K, Peter HH, Lebrecht D (2012). Impact of rituximab on immunoglobulin concentrations and B cell numbers after cyclophosphamide treatment in patients with ANCA-associated vasculitides. PLoS One.

[9] Yates M, Watts RA, Bajema IM, Cid MC, Crestani B, Hauser T, et al. EULAR/ERA-EDTA recommendations for the management of ANCA-associated vasculitis. Ann Rheum Dis. 2016;23:2016;75(9):1583–94. 10.1136/annrheumdis-2016-209133 27338776

[10] Venhoff N, Niessen L, Kreuzaler M, Rolink AG, Hässler F, Rizzi M (2014). Reconstitution of the peripheral B lymphocyte compartment in patients with ANCA-associated vasculitides treated with rituximab for relapsing or refractory disease. Autoimmunity.

[11] Masi AT, Hunder GG, Lie JT, Michel BA, Bloch DA, Arend WP (1990). The American College of Rheumatology 1990 criteria for the classification of Churg-Strauss syndrome (allergic granulomatosis and angiitis). Arthritis Rheum.

[12] Leavitt RY, Fauci AS, Bloch DA, Michel BA, Hunder GG, Arend WP, et al. The American College of Rheumatology 1990 criteria for the classification of Wegener's granulomatosis. 1990; 33(8):1101–7. 10.1002/art.1780330807 2202308

[13] Jones RB, Ferraro AJ, Chaudhry AN, Brogan P, Salama AD, Smith KGC (2009). A multicenter survey of rituximab therapy for refractory antineutrophil cytoplasmic antibody–associated vasculitis. Arthritis Rheum.

[14] Smith RM, Jones RB, Jayne DR (2012). Progress in treatment of ANCA-associated vasculitis. Arthritis Res Ther.

[15] Smith RM, Jones RB, Guerry MJ, Laurino S, Catapano F, Chaudhry A (2012). Rituximab for remission maintenance in relapsing antineutrophil cytoplasmic antibody-associated vasculitis. Arthritis Rheum.

[16] Smith KG, Jones RB, Burns SM, Jayne DR (2006). Long-term comparison of rituximab treatment for refractory systemic lupus erythematosus and vasculitis: remission, relapse, and re-treatment. Arthritis Rheum.

[17] Tamura N, Matsudaira R, Hirashima M, Ikeda M, Tajima M, Nawata M (2007). Two cases of refractory Wegener's granulomatosis successfully treated with rituximab. Intern Med.

[18] Breedveld F, Agarwal S, Yin M, Ren S, Li NF, Shaw TM (2007). Rituximab pharmacokinetics in patients with rheumatoid arthritis: B-cell levels do not correlate with clinical response. J Clin Pharmacol.

[19] Vögelin M, Biedermann L, Frei P, Vavricka SR, Scharl S, Zeitz J (2016). The impact of azazioprine-associated lymphopenia on the onset of opportunistic infections in patients with inflammatory bowel disease. PLoS One.

[20] Al Rifai A, Prasad N, Shuttleworth E, McBurney H, Pushpakom S, Robinson A (2011). Natural history of azathioprine-associated lymphopenia in inflammatory bowel disease patients: a prospective observational study. Eur J Gastroenterol Hepatol.

[21] Roberts RL, Barclay ML (2015). Update on thiopurine pharmacogenetics in inflammatory bowel disease. Pharmacogenomics.

